# Unsupervised fuzzy pattern discovery in gene expression data

**DOI:** 10.1186/1471-2105-12-S5-S5

**Published:** 2011-07-27

**Authors:** Gene PK Wu, Keith CC Chan, Andrew KC Wong

**Affiliations:** 1 Department of Computing, The Hong Kong Polytechnic University, Kowloon, Hong Kong; 2 Department of Systems Design Engineering, University of Waterloo, Waterloo, Ontario N2L 3G1, Canada

## Abstract

**Background:**

Discovering patterns from gene expression levels is regarded as a classification problem when tissue classes of the samples are given and solved as a discrete-data problem by discretizing the expression levels of each gene into intervals maximizing the interdependence between that gene and the class labels. However, when class information is unavailable, discovering gene expression patterns becomes difficult.

**Methods:**

For a gene pool with large number of genes, we first cluster the genes into smaller groups. In each group, we use the representative gene, one with highest interdependence with others in the group, to drive the discretization of the gene expression levels of other genes. Treating intervals as discrete events, association patterns of events can be discovered. If the gene groups obtained are crisp gene clusters, significant patterns overlapping different gene clusters cannot be found. This paper presents a new method of “fuzzifying” the crisp gene clusters to overcome such problem.

**Results:**

To evaluate the effectiveness of our approach, we first apply the above described procedure on a synthetic data set and then a gene expression data set with known class labels. The class labels are not being used in both analyses but used later as the ground truth in a classificatory problem for assessing the algorithm’s effectiveness in fuzzy gene clustering and discretization. The results show the efficacy of the proposed method. The existence of correlation among continuous valued gene expression levels suggests that certain genes in the gene groups have high interdependence with other genes in the group. Fuzzification of a crisp gene cluster allows the cluster to take in genes from other clusters so that overlapping relationship among gene clusters could be uncovered. Hence, previously unknown hidden patterns resided in overlapping gene clusters are discovered. From the experimental results, the high order patterns discovered reveal multiple gene interaction patterns in cancerous tissues not found in normal tissues. It was also found that for the colon cancer experiment, 70% of the top patterns and most of the discriminative patterns between cancerous and normal tissues are among those spanning across different crisp gene clusters.

**Conclusions:**

We show that the proposed method for analyzing the error-prone microarray is effective even without the presence of tissue class information. A unified framework is presented, allowing fast and accurate pattern discovery for gene expression data. For a large gene set, to discover a comprehensive set of patterns, gene clustering, gene expression discretization and gene cluster fuzzification are absolutely necessary.

## Background

In the recent years, with the progress of microarray technology, the parallel execution of microarray experiments on a very large set of genes accelerates investigations in many ways. Microarray chips are used to calibrate changes in gene expression levels and for classifying gene groups. Their data represented by expression tables consist of rows of genes and columns of samples. Many potential applications of gene expression data analysis such as medical diagnosis, gene function prediction, cancer classification, etc as technology progresses [1] are becoming more and more important.

In microarray gene expression data analysis, many approaches have been proposed. They can be grouped into supervised approach [2-5] and unsupervised approach [6-8]. The goal of the former is to build classifiers from a set of pre-classified samples and use them for classificatory purposes while that of the latter is to group similar genes or samples into clusters. For some cases, the class information of genes could be questionable or unavailable. Thus, there is no reliable ground truth for supervised approach. Traditional unsupervised approaches include clustering 1) the genes, 2) the samples, and 3) both genes and samples simultaneously (known as co-clustering or bi-clustering). They attempt to uncover either how the expression of a specific gene affects the expression of other genes or how different genes are expressed as a whole relating to certain cellular conditions [10]. It is believed that if a gene is regulated by several transcription factors based on the organism condition, the regulatory patterns may span several gene groups. Recently, a fuzzy approach to cluster genes was proposed [15]. Though it is an effective technique for gene clustering and selections, it needs prior knowledge of tissue class for discretizing the gene expression levels before significant patterns of gene expressions could be found. Association rule mining is also applied to gene expression data analysis [9] where classificatory knowledge is not required for identifying frequent itemsets. However, its weakness is the difficulty in deciding the minimum support and minimum confidence for associations to be considered interesting and also the discretization method in binning the gene expression levels to “up”, “down” or “up nor down” by a threshold value which may cause a significant loss of important information. To discover statistically significant association patterns as reported in [10] for better discretization of gene expression levels tissue class information is still required before pattern discovery.

In [11], a new method known as MACA which stands for **M**ixed-Mode **A**ttribute **C**lustering **A**lgorithm was proposed for discovering patterns on large mixed-mode data sets without relying on prior classificatory knowledge. A mixed-mode data set is referred to one that contains numeric, symbolic or categorical data. MACA can also be applied to continuous valued data by converting the continuous data into interval events. Hence, it brings out the association patterns among genes explicitly. To apply this to gene expression data analysis, each gene is considered as an attribute and each sample a tuple. Thus, for a very large data set, we will apply MACA which maximizes the interdependence among attributes (genes) within attribute clusters (groups) [11] to break down the gene pool into optimal crisp attribute clusters. However, there is still a drawback: it will miss some significant patterns that may overlap different crisp attribute clusters. This paper which extends the work in [11] comes up with a new extension known as FMACA - **F**uzzy **M**ixed-Mode **A**ttribute **C**lustering **A**lgorithm to allow overlapping relationship to be found among attribute clusters by MACA. Thus, patterns span across crisp attribute clusters could be discovered within the overlapping or fuzzy attribute clusters.

Treating each gene as an attribute and its expression levels as the attribute value, genes and attributes are equivalent in this paper. To evaluate the effectiveness of our method, we apply it first on a synthetic data set to illustrate the necessity and capability of the proposed algorithm and then on a gene expression data set, both with their known class label removed. After fuzzy gene clustering and data discretization, we bring back the class labels to the data set and assess the strength of the association patterns discovered through the classification performance using the patterns and rules discovered from the discretized interval events obtained not based on class labels.

## Methods

### Mixed mode attribute clustering

Consider a gene expression data set *T* containing a set of gene samples. |*T*| (the cardinality of *T*) is the total number of gene samples. Every sample (tuple), *t* ε *T*, is described by *N* gene expression levels (attributes) represented by *G* = {*G*_1_, …, *G_N_*}. Each *G_i_* , 1 ≤ *i* ≤ *N* can be seen as a continuous random variable. Thus, a realization of *G* can be denoted by *g_k_* = {*g*_1_*_k_*, … , *g_ik_* , … , *g_Nk_*} where { *g_ik_* | 1 ≤ *i* ≤ *N*} can assume any value in the domain of *G_i_* , *dom*(*G_i_*) ⊆ ℜ, where ℜ is the real number. Thus, each sample, *t* ε *T*, in the gene expression data set is a realization of *G*.

Here, we first employ our **M**ixed-Mode **A**ttribute **C**lustering **A**lgorithm (MACA) [11] to cluster genes (attributes). MACA is evolved from the **A**ttribute **C**lustering **A**lgorithm (ACA) [12]. It requires continuous valued data to be first discretized using class information. MACA can be conducted utilizing the correlation between attributes without relying on given class information. Thus, meaningful gene (attribute) clusters could be found by MACA such that genes within a gene (attribute) cluster have high interdependence with each other, whereas genes in different gene (attribute) clusters are less correlated. MACA uses a normalized redundancy measure(1)

to account for interdependence between genes where *I*(*G_i_*:*G_j_*) is the mutual information between *G_i_* and *G_j_*, and *H*(*G_i_*:*G_j_*) is the joint entropy of *G_i_* and *G_j_*. To compute *R* between continuous valued data, we use a contingency table with as many bins as possible to estimate the probability density function. Let |*T|* be the sample size of the gene expression data set, *m* be the number of bins and α be the least number of data points in a cell. In practice, α is the parameter chosen in the rule of thumb manner (say 2 or 3), ensuring that each cell in the contingency table will have at least α data points. Thus, the number of bins is calculated as:

Once *m* is decided for *G_i_*, 1 ≤ *i* ≤ *N*, the gene expression levels can be partitioned into *m* intervals and thus treated as discrete valued attributes. Therefore, *I*, *H* and *R* can then be computed.

MACA is based on the *k*-modes algorithm of ACA that finds disjoint attribute clusters. Evolved from the *k*-means algorithm, it uses a) the mode representing the attribute with highest interdependence with other attributes in the attribute cluster instead of the mean with minimum sum of distances among samples in the cluster and b) the normalized interdependence redundancy measure *R* between attributes instead of the Euclidean distance between samples in the *k*-means algorithm. The mode denoted by *G^r^* is the most representative gene in gene cluster *r* found by:

*MR*(*G_i_*)≥ *MR*(*G_j_*) for all *j*∈{1,…,*p*}, *i* ≠ *j* (2)

where(3)

is the multiple interdependence redundancy measure *MR*[12] of *G_i_* within the gene cluster *r* with *p* genes.

In MACA, we use the *k*-modes ACA to obtain *k* attribute clusters iteratively. In each iterative round, we identify the mode *G^r^* of each attribute cluster and compute *SR* which is the sum of *MR* of the mode from all the *k* attribute clusters as:(4)

By selecting *k* such that(5)

MACA then renders the *k* attribute clusters to be considered as a local optimal cluster configuration.

### Attribute cluster fuzzification

Now from the *k* gene clusters obtained, each gene cluster *C_r_*, *r*∈{1,… ,*k*} contains a representative gene, *G^r^*. After the gene clustering, every gene *G_i_*, *i*∈{1,… ,*N*} is assigned to only 1 gene cluster *C_r_*∈{*C*_1_,… ,*C_k_*}, where the gene clusters are disjoint, i.e. *C_r_ ∩ C_r_*= Ø for all *s* ∈{1,…,*k*} - {*r*}. However, if situations arise that a gene may have strong correlation to more than one gene clusters or that an association pattern among a collection of gene samples might overlap different gene clusters, they may not be found by our method at this phase. Hence we move on to the second phase that is to fuzzify a crisp gene cluster to encompass genes from other gene clusters if those genes have fuzzy characteristic function (in terms of correlation) to the crisp gene cluster. This procedure makes each gene bear varying degrees of fuzzy membership to other gene clusters such that high-order patterns overlapping crisp gene clusters could be discovered.

To construct the fuzzy membership, *R* in Equation (1) is adopted to derive a fuzzy interdependence redundancy measure [15]. Given that each gene is having a certain *R* to the mode of each gene cluster, we define a degree of fuzzy membership of a gene as the fractional part of the total possible membership assigned to the current gene cluster as.(6)

where *μ_r_*(*G_i_*) is the fuzzy membership function that returns the degree of membership of gene *i* in gene cluster *r;k* is the optimal number of gene clusters; *m* is the fuzzification parameter; *R*(*G_i_*:*G^c^*) is the interdependence redundancy between gene *i* and the mode of gene cluster *c*; and *R*(*G_i_*:*G^r^*) is the interdependence redundancy between gene *i* and the mode of gene cluster *r*. It has been shown that the following property (Equation 7) is desirable for the stability of fuzzy logic controllers [16,17](7)

The fuzzification parameter *f* is a real number > 1 for normalizing and fuzzifying the measure. For *f* = 2, this means to normalize the measure linearly to make their sum 1. For *f* close to 1, the gene closest to the representative gene is given more weight than others. With the fuzzy membership function defined, we can consider the correlation of each gene among the entire group of gene clusters.

## Continuous data discretization

Using the information extracted by attribute clustering and fuzzification, this phase is an important step towards pattern discovery within a fuzzy gene cluster. It involves discretizing the domains of gene expression levels by maximizing the interdependence between the gene expression levels and the representative genes within each fuzzy cluster.

We first employ Optimal Class-Dependence Discretization (OCDD) [14] to partition the gene expression levels of each gene into a finite number of intervals. Treating the representative gene (the mode) as the class attribute in each gene group, the mode is first discretized. In general, if the number of intervals is decided, in view of no other information, entropy maximization is used for discretizing the mode. In this paper, due to the relatively small sample size, we select 3 intervals. We can label them as 3 states: “highly expressed” (H), “normally expressed” (N) and “lowly expressed” (L). Once the mode that is the most representative gene in each fuzzy cluster is partitioned, each gene other than modes can be partitioned by OCDD considering the mode as the class label. Using the mode to drive the discretization, each gene is partitioned multi-times - each time with a different fuzzy gene group. Each partitioning result is associated with a degree of membership to a gene group.

After all gene expression levels are discretized into a finite number of intervals, the gene expression data set contains only categorical data and the pattern discovery phase can be conducted.

### Discovery of statistically significant patterns

In this phase, pattern discovery [18] method for categorical data could be applied readily. In an unsupervised manner, it detects high-order patterns defined as statistically significant associations of 2 or more primary events from different attributes using the adjusted residuals *d* to test the significance of its occurrence against the independence assumption [18]. The *adjusted residual* is a normalized statistical measure that accounts for the deviation of the observed frequency of an association (order > 2, i.e. number of attributes > 2) from its expected default model of independence [18]. An example 3rd-order pattern for the gene expression data set is {*G_x_* = [*g_ix_*, *g_ix_*], *G_y_* = [*g_iy_*, *g_iy_*], *G_z_* = [*g_iz_*, *g_iz_*]} with an adjusted residual of a certain value. It is interpreted as a 3rd-order pattern containing statistically significant associations of 3 primary events - *G_x_* = [*g_ix_*, *g_ix_*], *G_y_* = [*g_iy_*, *g_iy_*], *G_z_* = [*g_iz_*, *g_iz_*] from 3 attributes - *G_x_*, *G_y_* and *G_z_*. If the association pattern is conditioned by the class attribute, it can be treated as a classification rule [19], i.e. if {antecedent or left-hand-side or LHS} then {consequent or right-hand-side or RHS}. The *weight of evidence* measure *WofE* in information theory [19] is used to quantify the evidence of the joined significant association rules to support or against a certain class membership. An example rule for the gene expression data set is if {*G_x_* = [*g_ix_*, *g_ix_*] and *G_y_* = [*g_iy_*, *g_iy_*]} then {“Normal”} with a weight of evidence of a certain value.

## Results

### Synthetic data set

**Figure 1:**
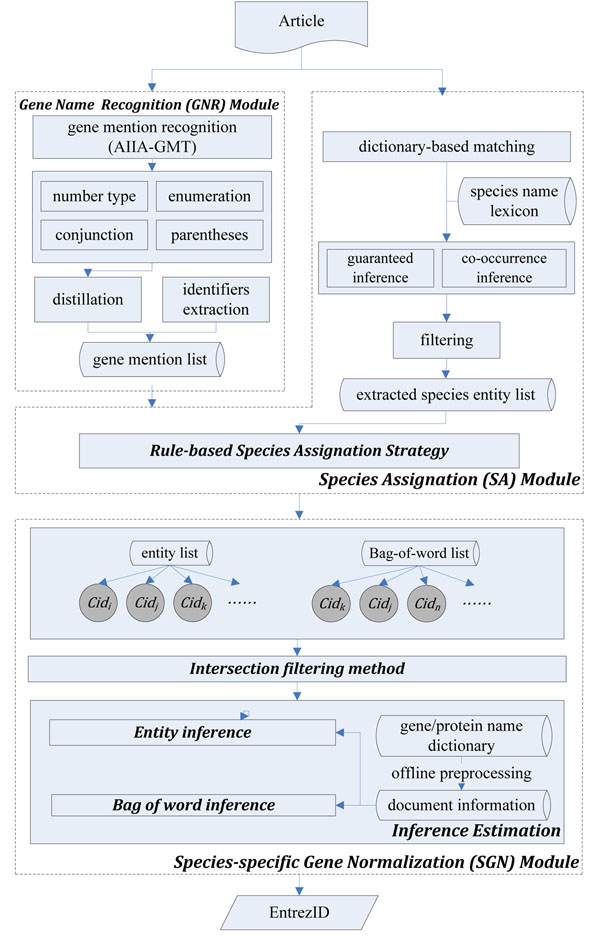
**Attributes of the synthetic data set** A diagram illustrating the attributes of the synthetic data set in their corresponding attribute groups. There are 3 attribute groups. The attribute with yellow circle is the mode of the attribute group. The dashed circle indicates the overlapping relationship of attributes in fuzzy attribute clusters.

The synthetic data set is designed to show the necessity and the capability of our proposed method. It is composed of 20 attributes: 5 discrete and 15 continuous (Figure 1). Let a set of attributes be denoted as *A_1_*, *…*, *A_20_. A*_1_ and *A*_2_ are discrete attributes which can take on a value from alphabets {“T*”*, “F*”*}*. A*_3_, *A*_4_, and *A*_5_ are discrete attributes taking on a value from alphabets {“X*”*, *“*Y*”*, *“*Z*”*}*. A*_6_, *…*, *A*_20_ are continuous attributes taking on values in {0 ≤ ℜ ≤ 1} where ℜ is a real number.

**Figure 2:**
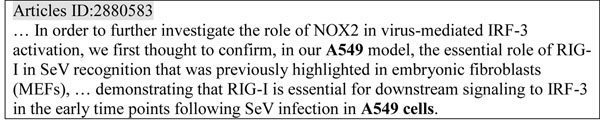
**Imposition of intrinsic classes by adjusting the attribute values of certain attributes** A diagram illustrating the class membership (*C*_1_, ... , *C*_5_) by imposing the values of *A*_1_, *A*_6_ and *A*_13_.

Each tuple is pre-classified into 1 of the 5 classes: *C*_1_, *…*, *C*_5_ by imposing the values of *A*_1_, *A*_6_ and *A*_13_ among the tuples (Figure 2) for defining the class membership. For overlapping attribute cluster relationship, *A*_4_, *A*_5_ and *A*_6_ are associated with both attribute cluster 1 and 3 with different degrees of membership. From Figure 1, we observe that *A*_6_ is the mode of attribute cluster 3, *AC*_3_, and *μ*_*AC*_1__ (*A*_6_) >*μ*_*AC*_2__(*A*_6_). *A*_1_ and *A*_13_ is the mode of attribute cluster 1, *AC*_1_, and attribute cluster 2, *AC*_2_, respectively. The attribute values are generated in the following manner.

*A_2_*: “T” if *A*_13_ < 0.2; “F”, otherwise.

*A*_3_: “X” if *A*_13_ < 0.2; “Y” if 0.2 ≤ *A*_13_ < 0.4; “Z”, otherwise.

*A*_4_: “X” if *A*_6_ < 0.3; “Y” if 0.3 ≤ *A*_6_ < 0.6; “Z”, otherwise.

*A*_5_: “Y” if *A*_6_ < 0.2; “Z” if 0.2 ≤ *A*_6_ < 0.4; “X”, otherwise.

*A_6_*: uniformly distributed within [0, 0.7] if *A*_1_ = “T” and *A*_13_ < 0.5; uniformly distributed within (0.3, 0.8] if *A*_1_ = “T” and *A*_13_ >= 0.5; uniformly distributed within [0, 1], otherwise.

*A*_7_: uniformly distributed within [0, 0.5] if *A*_1_ = “T”; uniformly distributed within (0.5, 1], otherwise.

*A*_8-12_: uniformly distributed within [0, 0.5] if *A*_1_ = “F”; uniformly distributed within (0.5, 1], otherwise.

*A*_14-17_: uniformly distributed within [0, 0.3) if *A*_13_ < 0.3; uniformly distributed within [0.3, 0.6) if 0.3 ≤ *A*_13_ < 0.6; uniformly distributed within [0.6, 1], otherwise.

*A*_18-20_: uniformly distributed within [0.3, 0.6) if *A*_13_ < 0.3; uniformly distributed within [0.6, 1] if 0.3 ≤ *A*_13_ < 0.6; uniformly distributed within [0, 0.3), otherwise.

In our experiment, 1800 tuples of mixed mode attributes are generated. *C*_1_, *…*, *C*_5_ contain 500, 300, 300, 400 and 300 tuples respectively. For practicality, 25% noise is added to the data by replacing 450 tuples with random values. We first use Mixed Mode ACA (MACA) to obtain attribute clusters, cluster modes and optimal cluster configuration *k* in order to reveal the correlated relationship. Then, in order to reveal the overlapping relationship, we use Fuzzy Mixed Mode ACA (FMACA), with fuzzification parameter *f* = 1.5 to obtain the degree of membership of each attribute *A_i_* to each attribute cluster *AC_j_*_,_.

As shown in Table 1, MACA reveals the attribute grouping without prior knowledge (class label excluded). It is worth to note that without fuzzification, however, it cannot show how some attributes are related among different attribute clusters since an attribute is a member of only one attribute cluster. By FMACA, it shows that 3 attributes (*A*_4_, *A*_5_ and *A*_6_) are indeed overlapping with *AC*_1_, *AC*_2_ and *AC*_3_ with different degrees of membership (*μ*_1_(*A*_4_) = 8.9602%, *μ*_1_(*A*_5_) = 2.4429%, *μ*_1_(*A*_6_) = 0.3961%, *μ*_2_(*A*_4_) = 0.0005%, *μ*_2_(*A*_5_) = 0.0031%, *μ*_2_(*A*_6_) = 0.0001%, *μ*_3_(*A*_4_) = 91.0393%, *μ*_3_(*A*_5_) = 97.5540%, *μ*_3_(*A*_5_) = 99.6037%).

**Table 1:** **Attribute clusters discovered by MACA** The items in each attribute cluster are ranked by their *MR*.

AC	M	SR	Item
**1**	*A*_1_	1.7159	*A*_1_, *A*_8_, *A*_7_, *A*_11_, *A*_12_, *A*_10_, *A*_9_
**2**	*A*_13_	1.0494	*A*_13_, *A*_2_, *A*_3_, *A*_16_, *A*_15_, *A*_17_, *A*_14_, *A*_18_, *A*_20_, *A*_19_
**3**	*A*_6_	0.5978	*A*_6_, *A*_4_, *A*_5_

Key: AC – Attribute Cluster; M – Mode; SR - Sum of the Multiple Significant Interdependence Redundancy Measure.

From this experiment and result, we realize that MACA is able to handle mixed mode data for effectively grouping of correlated attributes while FMACA, in addition, uncovers the overlapping relationship of each attribute to different attribute groups.

### Colon-cancer gene expression data set

We next apply FMACA to a colon-cancer gene expression data set. The colon-cancer data set [13] (62 samples and 2000 genes) is chosen to be analyzed due to its public availability. In the data set, each sample (tuple) is pre-classified into either normal or cancerous.

Since our method is unsupervised, we remove the tissue class label of samples in the initial experimental phase. We first cluster the genes to obtain the gene groups (clusters). As our FMACA supports mixed mode data, it is unnecessary to discretize the continuous data initially. As expected, FMACA found 7 optimal gene groups, the same result as reported by [12]. The result shows that our pattern discovery is able to uncover the correlated genes (attributes) and patterns without using class information. The top 5 genes of the 7 discovered gene groups includes 1)H05814, X02874, U33429, H22579, H25940, 2)T73092, R26146, T90851, R93337, T69446, 3)M26383, U34252, T59162, M27749, T54341, 4)T51849, D13243, X52008, R48936, X14968, 5)T90036, R81170, X67235, L20469, T63133, 6)T92451, H11460, H23975, R70030, D10522, 7)H71627, X74795, T55840, D17400, R71585. The top 1 gene of each gene group is the mode (most representative gene). These 35 genes are selected for classification in the second experimental phase.

In the second experimental phase, we first discretize each mode into 3 intervals by entropy maximization and then discretize the other genes by OCDD. After all genes are discretized, we put back the tissue class labels as an attribute to the data set. This preprocessed data, which is processed in an unsupervised manner, is trained by popular classification methods for building classifiers. We compare our results with those reported in [12].

The classification rate using 1) C5.0 and 2) our pattern discovery with data preprocessed by ours is 85.48% and 91.94% respectively while those as reported by [12], which preprocessed the data in a supervised manner, is 91.9% and 100% respectively. It shows that the proposed method is comparable to that requiring prior class information. The more significant implication is that even without class labels, the intrinsic interdependence of gene expression levels are brought forth: 1) to reveal the inherent relationship of the gene groups, 2) to select the most representative genes in each group, 3) to use their combined relationship to relate back to the class relation to achieve a high classification rate and 4) to use a fuzzy membership function to weigh the overlapping attributes so as to detect a more comprehensive set of patterns. As a consequence, the discretized data driven by inherent relationship to render high classification results evidences the efficacy of the proposed method.

**Figure 3:**
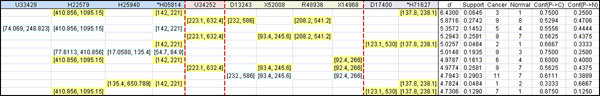
**Top 10 patterns of colon cancer data set** Different gene groups are filled in different colors and are separated by dashed lines. Patterns highlighted with yellow color indicate genes spanning across different gene groups while patterns highlighted with light blue color indicate genes in the same gene group. *d* is the adjusted residual. Conf(P->C) is the confidence that for a certain percentage of the samples containing the discovered pattern is cancerous. Conf(P->N) is the confidence that for a certain percentage of the samples containing the discovered pattern is normal. The gene marked with "*" indicates it is the mode of the gene group.

**Figure 4:**
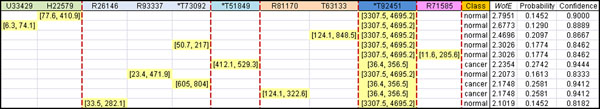
**Top 10 rules colon cancer data set** Different gene groups are filled in different colors and are separated by dashed lines. LHS of each rule is the gene expression intervals and the RHS is the class. *WofE* is the weight of evidence measure. The gene marked with "*" indicates it is the mode of the gene group.

To show the transparency of our system, we here provide some patterns and rules for reference and further discussion. Top 10 patterns and rules are shown in Figure 3 and Figure 4 respectively. Some of them are listed here for illustration. Pattern 1 is {H22579 = [410.9, 1095.2], H05814 = [137.5, 557.4], H71627 = [100.2, 467.3]} with an adjusted residual of 6.43. Pattern 2 is {U34252 = [223.1, 632.4], D13243 = [232, 586], R48936 = [208.2, 541.2]} with an adjusted residual of 5.87. Pattern 3 is {U33429 = [74.1, 248.8], H22579 = [410.9, 1095.2], H05814 = [142, 221]} with an adjusted residual of 5.36. Rule 1 is if {(H22579 = [77.6, 410.9] and T92451 = [3307.5, 4695.2]} then {“Normal”} with a weight of evidence of 2.7951. Rule 2 is if {(U33429 = [6.3, 74.1] and T92451 = [3307.5, 4695.2]} then {“Normal”} with a weight of evidence of 2.6773. Rule 3 is if {(T63133 = [124.1, 848.5] and T92451 = [3307.5, 4695.2]} then {“Normal”} with a weight of evidence of 2.4696.

Among the top 10 patterns and rules, we observe that some are composed of genes spanning across different gene groups. It reflects the usefulness of gene cluster fuzzification. Without it, some significant patterns will not be uncovered. For instance, 7 patterns out of the top 10 contain genes from different gene groups. From the rules discovered, we also believe that genes spanning across gene groups are important. In Figure 4, it shows that the probabilities of some rules’ occurrences are low but, in contrast, these rules, which have high weight of evidences as discovered by our approach, have high values of confidence. It means that the probability of finding the RHS of the rule in the colon cancer data set under the condition that these gene samples also contain LHS is high.

## Conclusions

We have shown that the proposed method for analyzing the error-prone microarray is effective even without the presence of tissue class information. Here we would like to highlight several key points stressing the significance of our proposed methodology. First, even without class information, our method is effective for analyzing the error-prone microarray data. Second, the existence of correlation among continuous valued gene expression levels suggests members in the gene groups have high interdependence. Third, overlapping relationship among attribute clusters could be uncovered through cluster fuzzification. Forth, previously unknown hidden patterns residing in overlapping attribute clusters can be discovered in the fuzzy attribute clusters. Furthermore, the discovered high order patterns reveal multiple gene interaction patterns in cancerous tissues not found in normal tissues.

From the experimental results, we observe that to discovery a comprehensive set of patterns for a large gene set, gene clustering, gene expression discretization and gene cluster fuzzification are absolutely necessary. Attribute clustering enables us to partition a large gene set (2000 for colon cancers) into correlated subsets, making selection of representative genes from each subset more meaningful and effective. Discovering patterns from fuzzy attribute clusters allows us to find those patterns spanning across different crisp attribute groups. As revealed in our colon cancer data experiment, without fuzzification, we may miss 70% of the significant patterns spanning across gene groups and also the high order patterns associated with different tissue classes. In conclusion, this paper renders a unified framework which allows fast and accurate pattern discovery for gene expression data - an important computational step closer to meeting the challenge of discovering new biological knowledge from biological data.

## List of abbreviations used

***R***: Interdependence redundancy measure; ***MR***: Multiple interdependence redundancy measure; ***SR***: Sum of multiple significant interdependence redundancy measure; ***ACA***: Attribute clustering algorithm; ***MACA***: Mixed-mode attribute clustering algorithm; ***FMACA***: Fuzzy mixed-mode attribute clustering algorithm.

## Competing interests

The authors declare that they have no competing interests.

## Authors' contributions

GPKW carried out the pattern discovery studies, participated in the experiments and drafted the manuscript. KCCC conceived of the study and participated in its design. AKCW participated in the design and coordination and helped to draft the manuscript. All authors read and approved the final manuscript.
